# Law of Missouri Concerning the Practice of Medicine

**Published:** 1874-09

**Authors:** 


Law of Missouri Concerning the Practice of Medicine.—We furnish below a synopsis of the law passed at the last session of the Legislature of Missouri in relation to the practice of medicine and surgery in this State, approved March 27, 1874:

1. It is made unlawful for any person to practice medicine or surgery in this State, who shall not have first received the degree of doctor of medicine from some medical college or university duly established under and by virtue of the laws of the State or Country in which the same is situated.

2. Every person wishing to commence the practice of medicine or surgery in this State, is required to file a copy of his diploma in the office of the County Clerk of the county in which he resides, to be sworn to and subscribed by the party filing the same; and thereupon the County Clerk registers the diploma, for which a fee must be paid.

3. Persons practicing, or who shall commence to practice before the first of September, 1874, shall register without diplomas.

4. The penalty for non-compliance with the act, upon conviction, is fixed at not less than twenty-five nor more than five hundred dollars; and the fact that such person has not filed his diploma is a sufficient defence in any action which he may bring to recover his fees for professional services.

